# The roles of neural stem cells in myelin regeneration and repair therapy after spinal cord injury

**DOI:** 10.1186/s13287-024-03825-x

**Published:** 2024-07-08

**Authors:** Chun Li, Yuping Luo, Siguang Li

**Affiliations:** 1 Key Laboratory of Spine and Spinal Cord Injury Repair and Regeneration of Ministry of Education, Department of Neurology, Tongji Hospital, Tongji University School of Medicine, Shanghai, 200065 China; 2 Tongji University Cancer Center, Shanghai Tenth People’s Hospital of Tongji University, Tongji University School of Medicine, Shanghai, 200092 China; 3 Stem Cell Translational Research Center, Tongji Hospital, Tongji University School of Medicine, Shanghai, 200065 China

**Keywords:** Spinal cord injury, Neural stem cells, Oligodendrocytes, Myelin regeneration, SCI repair therapy, NSC transplantation

## Abstract

Spinal cord injury (SCI) is a complex tissue injury that results in a wide range of physical deficits, including permanent or progressive disabilities of sensory, motor and autonomic functions. To date, limitations in current clinical treatment options can leave SCI patients with lifelong disabilities. There is an urgent need to develop new therapies for reconstructing the damaged spinal cord neuron-glia network and restoring connectivity with the supraspinal pathways. Neural stem cells (NSCs) possess the ability to self-renew and differentiate into neurons and neuroglia, including oligodendrocytes, which are cells responsible for the formation and maintenance of the myelin sheath and the regeneration of demyelinated axons. For these properties, NSCs are considered to be a promising cell source for rebuilding damaged neural circuits and promoting myelin regeneration. Over the past decade, transplantation of NSCs has been extensively tested in a variety of preclinical models of SCI. This review aims to highlight the pathophysiology of SCI and promote the understanding of the role of NSCs in SCI repair therapy and the current advances in pathological mechanism, pre-clinical studies, as well as clinical trials of SCI via NSC transplantation therapeutic strategy. Understanding and mastering these frontier updates will pave the way for establishing novel therapeutic strategies to improve the quality of recovery from SCI.

## Introduction of the current status of spinal cord injury (SCI)

Spinal cord injury (SCI) is a destructive neurological and pathological condition that leads to a loss of motor, sensory and autonomic functions below the injury site. The permanent or progressive disabilities it brings can have a devastating impact on individuals and a significant burden on the society [1–3]. Most common causes for traumatic SCI are traffic accidents, falls, sports injuries and violence. For the past 30 years (from 1990 to 2019), the incidence of SCI was 0.9 million cases with an estimated 6.2 million cases lived with disability. SCI rates increased substantially for global prevalence (from 74.2 to 87.1%), incidence (from 30.3 to 69.8%) and cases lived with disability (from 56.3 to 76.0%), basing on the data from the Global Burden of Diseases, Injuries, and Risk Factors Study (GBD) 2019 [4]. Despite considerable strides in trauma management and medical care, available treatments can only provide supportive relief for patients with lifelong disabilities since the lack of effective treatment options for this devastating disease [5–7].

## Pathophysiology of SCI

**Fig. 1:**
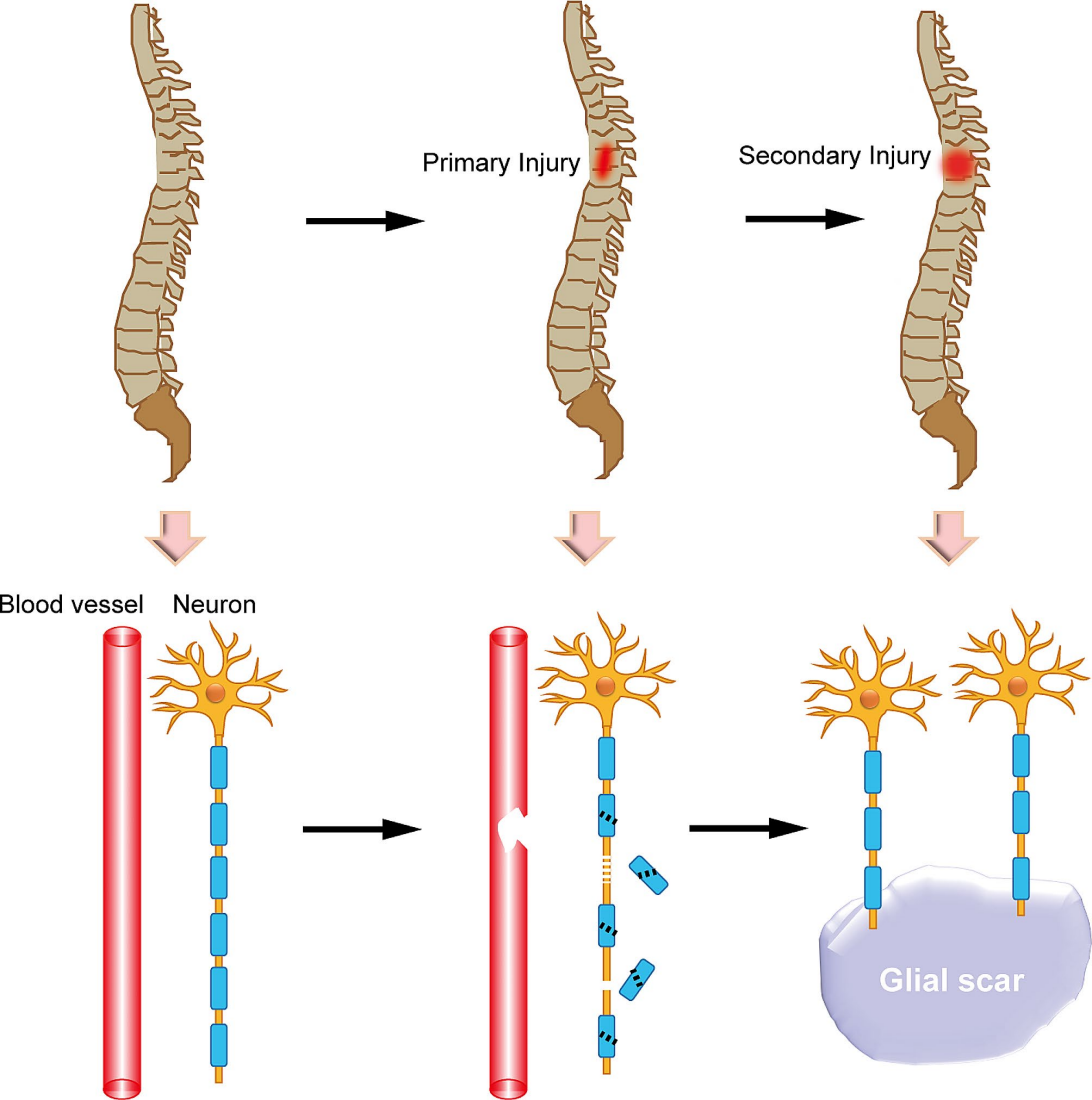
Primary and secondary injuries following the spinal cord injury. Primary injury results in disruption of the myelin sheath in the spinal cord, bone fragments and tear of spinal ligaments; secondary injury activates the inflammatory cascade, forms glial scars, and inhibits injury repair

SCI can be divided into primary injury and secondary injury according to the injury time and pathophysiologic changes [8, 9]. The initial stage is known as primary injury, which commonly occurs immediately after the injury caused by a sudden trauma, and is accompanied with bone fragments and tear of spinal ligaments. The secondary injury triggered by the primary injury, which produces further chemical and mechanical damage to spinal tissues, involving gliosis, glial scar, inflammation, demyelination and other reversible pathologic changes (Fig. 1) [2, 10].

The SCI pathophysiology of secondary injury comprises a complex cascade of interrelated events at the molecular, cellular and systemic levels [11, 12]. Injuries caused by trauma or other factors disrupt the structure of the spinal cord, leading to a breakdown of the blood-spinal cord barrier, and initiating an inflammatory response [13].

At the molecular level, the release of inflammatory cytokines, including interleukin (IL)-1a, IL-1b, IL-6, and tumor necrosis factor (TNF), recruits immune cells to the injury site and initiates the inflammatory cascade. This inflammatory response leads to further damage and cell death within the spinal cord [14, 15].

At the cellular level, disruption of blood vessels causes bleeding in spinal cord tissues, followed by invasion of neutrophils, monocytes, macrophages, T and B lymphocytic cells [14, 16]. In addition, glial cells involved in gliosis or the formation of glial scars undergo morphological and functional changes following SCI. Gliosis refers to the reactive response of astrocytes, microglia and other glial cells, to central nervous system (CNS) injury, which can include SCI, trauma, stroke, or neurodegenerative diseases [17, 18]. This response is characterized by the proliferation and hypertrophy of these cells, along with altered gene expression, which can lead to the formation of a glial scar. The glial scar is a complex structure composed of various cell types, including reactive astrocytes, NG2 glia, fibroblasts, microglia, etc. [19, 20]. The glial scar has both beneficial and detrimental effects on CNS repair. On the one hand, it can act as a physical barrier to wall off the injury site, preventing the spread of toxic molecules and curtailing inflammation, which is crucial for the initial stages of wound healing. Reactive astrocytes within the glial scar also secrete growth factors and cytokines that can support survival and regeneration of certain neurons [21]. On the other hand, the glial scar poses significant challenges to axonal regeneration and can contribute to demyelination. Key factors that limit regeneration include physical barrier, chemical inhibition, inflammatory environment, and oligodendrocyte dysfunction [22–25]. Specifically, the dense network of glial cells and extracellular matrix (ECM) proteins, such as chondroitin sulfate proteoglycans (CSPGs), can physically impede the growth of axons, which is critical for restoring neural connections after injury [21, 22]. CSPGs and other molecules within the ECM can release inhibitory signals that actively suppress axonal growth and neurite extension. These molecules can interact with neuronal cell surface receptors, such as the Nogo receptor, to suppress growth signals [23]. Prolonged activation of microglia and the release of pro-inflammatory cytokines can create a hostile environment for axonal growth and contribute to further demyelination and neuronal damage [24]. Additionally, glial scar formation can adversely affect the function of oligodendrocyte precursor cells (OPCs), which are responsible for myelination. This disruption can result in demyelination, which in turn can compromise axonal conduction and further deteriorate neuronal function [20, 25]. Moreover, neurons undergo Wallerian degeneration, characterized by axonal swelling and cytoskeletal rearrangements, leading to the breakdown and degeneration of axons and their innervation structures [26, 27].

At the systems level, SCI results in the disconnection of surviving neural elements, dysfunction of the neural circuits and loss of motor, sensory and autonomic function. The poor axon growth ability and inhibitory factors of scar axon regeneration leads to the failure in regeneration of spinal cord and reconstruction of neural circuits [28, 29]. Therefore, therapies primarily focus on how to promote axon regeneration, axon myelination and neural circuits reconstructing.

## The basic characteristics of neural stem cells (NSCs)

Neural stem cells (NSCs) are present in all major subdivisions of the CNS in adult mammals, including the spinal cord [30–32]. NSCs are essential for the maintenance of CNS functions during development and the regeneration of all CNS cell populations [33, 34]. They have indefinite self-renewal capacity and the ability to give rise to neural progenitor cells (NPCs). Additionally, they have the potential to continue developing into mature neural lineages, such as neurons, astrocytes and oligodendrocytes [35–37].

NSCs reside along the axis of CNS. The two major active brain regions for regenerating NSCs are the subventricular zone (SVZ) of the lateral ventricles and the subgranular zone (SGZ) of the hippocampal dentate gyrus [38, 39]. In addition, the fourth ventricle [40] and the central canal ependyma of the spinal cord also harbors endogenous populations of NSCs, which have the potential to generate neural lineages [41].

## Mature oligodendrocytes from NSCs are essential for myelination

**Fig. 2:**
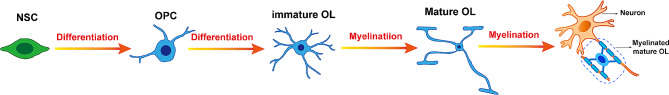
Schematic of oligodendroglial lineage depicting different developmental stages from neural stem cells (NSCs) to myelinate mature oligodendrocytes (OLs). Oligodendrocyte progenitor cells (OPCs) are capable to differentiate into immature oligodendrocytes (immature OLs), and then myelinate into mature OLs. The newly formed myelin wraps around axons, thus creating a new myelin sheath

Oligodendrocytes are one of the major glial cell types in the CNS besides astrocytes. During neural development, oligodendrocytes originate from NSCs and undergo a series of differentiation process to achieve a mature phenotype [42–45]. The maturation of oligodendrocytes is a prerequisite for the production and maintenance of the myelin sheath, which is a lipid-rich substance that wraps around axons to provide electrical insulation and support, thus highlighting the importance of oligodendrocyte lineage development for myelination (Fig. 2) [46–49]. Myelin is essential for the rapid transmission of electrical signals along axons and also provides trophic support to neurons [50–52]. Oligodendrocytes are distributed throughout the CNS, including the brain and spinal cord. They are critical for the normal functioning of the nervous system [53, 54]. The loss or damage of oligodendrocytes can lead to demyelination and neurodegeneration, causing various CNS diseases such as multiple sclerosis, acute disseminated encephalomyelitis, and other demyelinating diseases [55–57].

## Myelin regeneration process

SCI often results in damage to the myelin sheath, the insulating layer that surrounds nerve fibers and effectively transmits electrical signals [58]. Therefore, myelin regeneration is significant for the recovery process following SCI. Remyelination is the myelin regenerative response that follows demyelination, which restores saltatory conduction and function, maintains axon health, and is essential for the recovery from demyelinating diseases and injuries [59, 60]. NSC-derived oligodendrocytes have been shown to integrate into existing neural networks and to remyelinate damaged nerve fibers [61]. It is a complex process that involves the recruitment of NSC-derived oligodendrocyte precursor cells (OPCs) to the site of demyelination, their differentiation into mature oligodendrocytes and the formation of new myelin sheaths around axons [62, 63]. This process is controlled by a network of signaling molecules [64], transcription factors [65, 66] and microRNAs [67, 68].

## Endogenous NSCs neurogenesis after SCI

NSCs play a crucial role in remyelination after SCI. Given the right conditions, endogenous NSC niche in the SCI can be activated and start to proliferate rapidly in response to injuries. The inflammatory response following the injury releases signaling molecules and growth factors, which contribute to the activation and multiplication of NSCs [13, 69]. Subsequently, NSCs, guided by chemokines, growth factors and extracellular matrix, migrate from the NSC niche towards the injury site and the area requiring remyelination [70]. Once reaching the injury site, NSCs differentiate into oligodendrocytes, regulated by several signaling molecules such as Olig1/2 [71, 72], MBP [73, 74], PLP [75, 76] and MAG [77, 78]. The newly formed oligodendrocytes then produce myelin and wrap it around axons, thus creating a new myelin sheath [63]. While the neurogenesis of endogenous NSCs is a complicated and difficult process, considerable advancements are making strides towards leveraging this process for regenerative medicine treatments in SCI [79, 80].

## Potential sources of NSCs for transplantation strategies

Transplantation of NSCs has emerged as a potential therapeutic approach for SCI, aiming to replace lost cells, promote tissue repair and restore neural function. However, the identification of suitable sources for NSCs remains a critical issue. There are several potential sources of NSCs that can be used for transplantation strategies in SCI, including adult NSCs, embryonic stem cells (ESCs), induced pluripotent stem cells (iPSCs) and fetal neural progenitor cells (FNPCs) [81, 82]. They have the ability to self-renew and differentiate into neurons, astrocytes and oligodendrocytes.

## Adult NSCs

Transplantation of adult NSCs has shown beneficial effects in terms of availability, potency, immune rejection, ethical considerations and risk of tumorigenesis [83]. Originally, Reynolds and Weiss succeeded in isolating a population of cells from the striatum of adult mice that had the ability to proliferate and differentiate into neurons and glial cells in vitro, and formally proposed the concept of NSCs [84]. Later, Zhao and colleagues revealed that adult NSCs transplantation improved locomotor function in spinal cord transection rats, which associated with nerve regeneration and IGF-1R expression [85]. Recently, Fauser and colleagues successfully transplanted adult NSCs from midbrain periventricular regions into the hippocampal neurogenic niche [86]. Lv and colleagues evaluated the safety and efficacy of adult NSCs transplantation for cerebral palsy [87].

## Embryonic stem cells (ESCs)

ESCs are pluripotent stem cells derived from the inner cell mass of the blastocyst. They have the potential to self-renew and differentiate into any cell type in the body [88–90]. To prepare region-specific NPCs from ESCs for regenerative medicine researches, various in vitro directed methods have been established based on developmental stages that NSCs undergo during neural induction and differentiation in the CNS [81]. Initially, Bain and colleagues performed neural induction of mouse ESCs using embryoid body formation assay, and ESC-derived NSCs could differentiate into neurons capable of generating action potentials after RA treatment [91]. Subsequently, Chambers and colleagues proposed a novel method for generation of NPCs from ESCs by dual SMAD inhibition, which provided a more efficient approach for preparing NPCs from ESCs by remarkedly improving the conversion efficiency of ESCs to neural rosette and reducing the duration for neural induction [92]. Additionally, researchers described a straightforward approach for efficiently generating NSCs from ESCs using simplified medium formulations and procedures, indicating that dynamic changes of cell-substrate matrix interactions through short suspension culture period facilitated ectodermal lineage specification [93].

The differentiation of ESCs into NSCs offers a potential source of NSCs for transplantation strategies in SCI and other CNS disorders. However, ethical concerns and potential immunological rejection limit the use of ESCs in transplantation [94].

## Induced pluripotent stem cells (iPSCs)

iPSCs are artificially generated from reprogrammed somatic cells via the induction of pluripotent genes, and share the same developmental pluripotency as ESCs [95]. As such, they resolve the ethical controversy of ESCs, realize autologous cell transplantation and prevent immune rejection [96, 97].

In 2006, Yamanaka’s research team developed the reprograming technology to generate iPSCs from accessible somatic cells, thus ushering in a new era in translational and regenerative medicine [98]. Since similar properties of ESCs and iPSCs, iPSCs can share a similar approach with ESCs to the induction of NSCs. In 2014, D’Aiuto and colleagues reported a scalable protocol that allows robust and cost-effective generation of NSCs from iPSCs [99]. For the past decade, researchers have made great progress in stem cell therapies of iPSC-derived NSCs. Transplanting iPSC-derived NSCs improved CNS functional recovery after SCI in mice [100–105] and rats [106–108], by inhibiting demyelination and promoting synapse formation. In addition, Strnadel and colleagues reported the survival of iPSC-derived NSCs after spinal grafting in minipigs [109].

## Fetal neural progenitor cells (FNPCs)

FNPCs are neural progenitor cells derived from fetal tissue that have the ability to self-renew and differentiate into neural lineages. FNPCs have been used in transplantation strategies for SCI with promising results. Studies have shown their positive role in promoting tissue repair and functional recovery of the injured spinal cord [110, 111]. However, there are also potential risks that limit the use of FNPCs in clinical applications, such as immune rejection and ethical concerns surrounding the use of fetal tissue.

## Comparative evaluation of various sources of NSCs

In pre-clinical studies, several factors need to be considered when selecting the above sources of NSCs, including differentiation potential, integration and survival abilities, immunogenicity, ethical considerations, availability and scalability, safety and tumorigenicity, and functional outcomes.

Specifically, as for differentiation potential, ESCs and iPSCs are unrivaled, holding the theoretical capacity to differentiate into any cell type within the body, encompassing the full spectrum of neural lineages. Adult NSCs, while more limited in their plasticity, retain the ability to differentiate into a variety of neural cells, such as neurons, astrocytes, and oligodendrocytes. FNPCs exhibit multipotency as well, yet their differentiation potential is more constrained relative to ESCs and iPSCs [81, 111]. Regarding integration and survival abilities, adult NSCs and FNPCs, due to their closer developmental alignment with the host tissue in SCI, tend to integrate more effectively into the neural tissue. In contrast, ESCs and iPSCs necessitate meticulous differentiation to ensure proper integration and to prevent the risk of forming teratomas or other tumors [112]. In terms of immunogenicity, ESCs derived from non-autologous sources pose a higher risk of provoking an immune response, potentially leading to rejection. Autologous iPSCs, generated from an individual’s own cells, significantly mitigate this risk [113, 114]. Similarly, adult NSCs and FNPCs, particularly when autologous, present a reduced likelihood of immune rejection [111, 115]. From the ethical considerations, ESCs are derived from embryos, raising considerable ethical dilemmas [94]. Remarkably, iPSCs avoid these concerns by being generated from somatic cells and reprogrammed to a pluripotent state [96, 97]. Both adult NSCs and FNPCs are generally considered more ethically acceptable; however, it should be noted that FNPCs, derived from fetal tissue, may also engender ethical issues [112]. Concerning availability and scalability, iPSCs present the advantage of being scalable and the ability to be generated from any patient, which is highly promising for personalized medicine [114]. Adult NSCs, while more limited, can be isolated from various regions of the adult brain and expanded in vitro. Conversely, FNPCs face constraints due to the scarcity of available fetal tissue [116]. With respect to safety and tumorigenicity, adult NSCs are generally considered less likely to induce substantial gliosis, ascribed to their closer developmental congruence with the host tissue, while ESCs and iPSCs possess high differentiation potential and thus bear the inherent risk of gliosis if their differentiation process being misguided. ESCs and iPSCs carry a heightened risk of tumor formation and require meticulous differentiation approaches to mitigate this risk [94]. Adult NSCs and FNPCs are generally considered to have a reduced tumorigenic risk, favoring their use in certain applications [115]. With regard to functional outcomes, pre-clinical studies have shown that all of these mentioned cell types can contribute to a certain degree of functional improvement in animal models of SCI, with the magnitude of recovery being variable [85, 100, 110, 117, 118]. Factors such as the specific cell type, the timing and technique of transplantation, and the SCI model used are recognized to impact the outcomes.

## Advancements in NSC transplantation therapies for SCI treatment

**Fig. 3:**
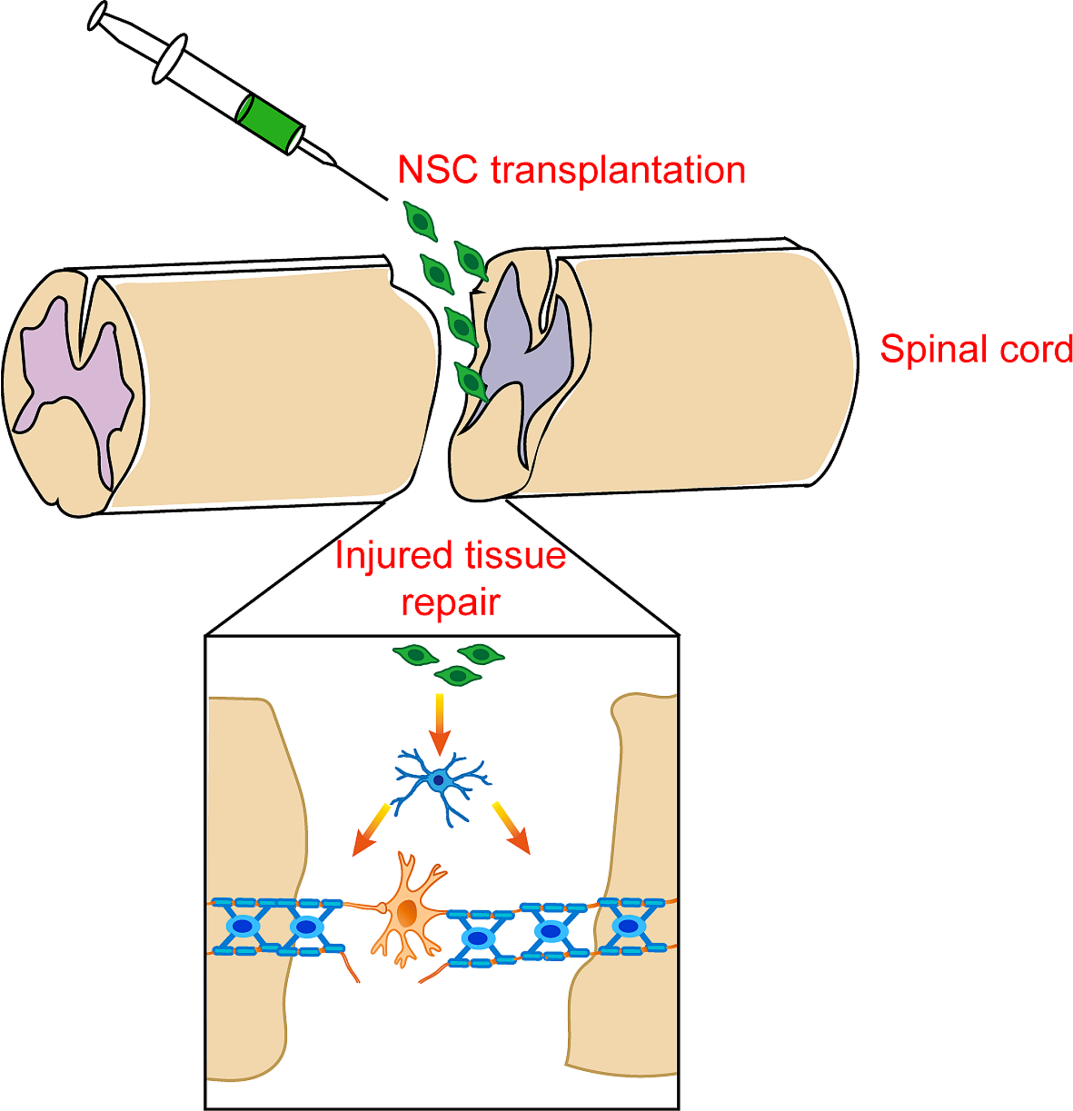
The transplantation of NSCs in the repair and recovery of injured spinal cord. After injecting into the injury site, NSCs rapidly proliferate and differentiate into oligodendrocytes. NSC-derived oligodendrocytes integrate into the destroyed neural networks and remyelinate damaged nerve fibers

NSC transplantation is a prospective therapeutic strategy for SCI. This approach aims to improve functional recovery by replacing damaged cells, restoring lost neural circuits, modifying SCI environment and promoting endogenous repair (Fig. 3) [119, 120]. With the development of regenerative medicine based on stem cell research, advanced novel strategies and technologies have been implemented in pre-clinical studies, some of which have already been applied in clinical trials [121, 122]. Nowadays, considerable progress has been made in pathological mechanism research, pre-clinical studies and clinical trials.

## Studies on pathological mechanism

In the past few decades, researchers have been attempting to fully elucidate the pathological mechanism of SCI, and to seek for effective strategies to promote axon regeneration and neural circuit remodeling; however, they have not made satisfactory achievements. Recently, single-cell sequencing technology and multi-omics analyses have been widely used in SCI research, providing a broader vision to elucidate pathological mechanisms of SCI [123–126].

A relatively early studies has shown that transplantation of NSCs together with administration of valproic acid dramatically enhanced the restoration of hind limb function, thus raising the possibility that epigenetic status in transplanted NSCs could be manipulated for providing effective treatment for SCI [127]. Another study revealed that NSC transplantation could modulate SCI-induced inflammatory responses and enhance neurological function after SCI via reducing M1 macrophage activation and infiltrating neutrophils [128].

Lately, researchers put forward that LncRNA-GAS5 promoted spinal cord repair and inhibited neuronal apoptosis through the transplantation of 3D-printed scaffold loaded with iPSC-derived NPCs [129]. STAT3 was identified as a target for NSCs to promote neuronal differentiation and functional recovery in rats with SCI [130]. Transplantation of Wnt4-modified NSCs improved inflammatory micro-environment of SCI by mediating M2 polarization [131]. Transplantation of NSC which preconditioned with hypoxia promoted SCI in rats by affecting transmembrane immunoglobulin domain-containing [132].

## Pre-clinical studies

Pre-clinical studies have shown that NSC transplantation can effectively promote spinal cord repair [133, 134]. In various animal models, NSCs have been successfully transplanted into injured spinal cords, and the therapeutic potential, safety and technical challenges of NSCs transplantation have been tested under multiple conditions [135–137]. These cells integrate with the host tissue, forming functional synapses and improving motor and sensory function. Pre-clinical studies have also evaluated different sources of NSCs, such as fetal spinal cord, adult brain and iPSCs. The use of iPSCs offers an attractive alternative since they can be derived from patients’ own cells, avoiding ethical issues and immune rejection [137].

In 1999, scientists first demonstrated the therapeutic potential of NSC transplantation in the context of SCI. The grafts, which derived from transplanting NSCs into a rat spinal cord, were observed to have the capacity to survive and differentiate into neurons, oligodendrocytes and astrocytes [138]. Since then, plenty of studies have shown that NSCs can be successfully transplanted into the injured spinal cord, and are beneficial to the functional recovery. Takano and colleagues showed that the neurotrophic factor HGF plays a key role in the enhanced functional recovery after NSC transplantation observed in aged mice with SCI [139]. Zhang and colleagues treated transplanted NSC with LiCl, and promoted the functional recovery in SCI rat [140]. Xue and colleagues found that transplantation of NSCs which preconditioning with high‑mobility group box 1 facilitated functional recovery after SCI in rats [141]. Kong and colleagues showed the effective recovery of acute SCI in mice via transplanting hiPSC-derived NSCs [103]. Ko and colleagues induced cellular differentiation of transplanted NSCs into neurons, and found that transplantation of neuron-inducing grafts positively charged gold nanoparticles for the treatment of SCI [142]. The pre-clinical studies on NSC transplantation therapy in SCI animal models are summarized in Table 1.

**Table 1:** Summary of pre-clinical studies on NSC transplantation therapy in SCI Animal models

Donor cells	Quantity of transplanted cells	Method of cell delivery	Time of transplantation	Outcomes	References
Human iPSC-derived-NSCs	1 × 10^5^	Injected into mouse spinal cord after laminectomy	about 1 month	Promoted functional recovery in mice by replacing missing neurons and attenuating fibrosis, glial scar formation, and inflammation	[103]
Fetal rat spinal cord-derived NSCs	1–4 × 10^5^	Transplanted into rat spinal cord at 9 days after contusion injury	5 weeks	Graft neurons extended processes into host tissue and formed synaptic formation with host neurons	[111]
Mouse ESCs- derived NSCs	Not mentioned	Transplanted into rat spinal cord 9 days after traumatic injury	2–5 weeks	Showed hindlimb weight support and partial hindlimb coordination	[138]
Mouse striata- derived NSCs	5 × 10^5^	Transplanted into mouse spinal cord after laminectomy	7 weeks	Showed the importance of neurotrophic factor HGF in the enhanced functional recovery	[139]
Rat spinal cord-derived NSCs	1 × 10^5^	Transplanted into rat spinal cord after injury	4 weeks	Showed that Lithium promoted survival and neuronal generation of grafted NSCs	[140]
Rat neocortices-derived NSCs	2 × 10^5^	Injected into rat spinal cord after injury	about 1 month	Showed that preconditioning with high‑mobility group box 1 facilitated functional recovery	[141]
Rat spinal cord-derived NSCs	4 × 10^5^	Injected into rat spinal cord after contusion injures	6 weeks	Suggested that neuron-inducing grafts embedding positively charged gold nanoparticles for the treatment of SCI	[142]
Fetal mouse spinal cord-derived NSCs	Not mentioned	Injected into mouse spinal cord immediately after dorsal column lesion	1–3 months	Graft neurons receive synaptic inputs from all host spinal cord tracts	[143]
Human embryonic spinal cord-derived NSCs	2 × 10^7^	Transplanted into monkey cervical after injury	2 weeks	Axon regeneration with synapse formation	[144]
Rat enteric nervous system-derived NSCs	1 × 10^6^	Injected into rat thoracic after drop weight contusion injury	3 days	Gastrointestinal tract could be a viable option for cell source	[145]

## Clinical trials

Numerous clinical trials on the stem cell treatment of SCI have been conducted worldwide [146–148], of which, several clinical trials are based on NSCs transplantation [149–151]. Due to the limitations of small sample sizes, variable trial designs and short follow-up durations in most studies, longer-term follow-up data are needed to evaluate the long-term safety and efficacy of NSC transplantation in the treatment of SCI [152].

Clinical trials have shown promising results, with some patients experiencing significant improvements in motor function and sensory perception following NSC transplantation. Research published in 2018 reported a first-in-human, phase I study of NSCs transplantation for chronic SCI. Unfortunately, this study is insufficient and debatable due to the lack of a control group and a small number of subjects, yet it paves the way for future research [153].

Several clinical trials have been conducted to evaluate the safety and efficacy of NSC transplantation in patients with SCI. A study published in 2019 showed clinical outcomes from a multi-center study of human NSC transplantation in chronic cervical SCI, aiming at assessing the safety and feasibility of NSC transplants for the treated participants [154]. Another study conducted in 2020 showed that the long-term results of the NSC transplants in spinal cords of 12 participants were reassuring. A six-year follow-up clinical assessment, containing sensory thresholds and neuroimaging augmenting, revealed short- and long-term surgical and medical safety for NSC transplantation therapy [155]. In 2021, researchers conducted a first-in-human clinical trial of transplantation of iPSC-derived NSCs in subacute complete SCI, with assessment of the safety of iPSC-derived NSC transplantation in patients and its impact on neurological function and quality-of-life outcomes [156]. Initial studies have demonstrated the safety of this approach, with no significant adverse events reported, however, results regarding efficacy have been ambiguous. Some studies have reported improvements in motor function and sensation, while others have not observed significant changes. The differences in outcomes may be attributed to factors such as source of transplanted cells, quantity of cells, and the time post-transplantation after injury [122, 157, 158]. The clinical trials on NSC transplantation therapy in SCI treatment are summarized in Table 2.

Despite the promising results of NSC transplantation in SCI treatment, there are still several challenges that need to be addressed. Future clinical trials should focus on optimizing cell sources, transplant techniques, and outcome measures to further enhance the therapeutic potential of NSC transplantation. The identification of appropriate patient populations and the development of combinatorial approaches that combine NSC transplantation with other regenerative strategies may also bring more effective treatment outcomes.

**Table 2:** Summary of clinical trials on NSC transplantation therapy in SCI treatment

Donor cells	Quantity of transplanted cells	Method of cell delivery	Outcomes	References
Human CNS derived-NSCs	2 × 10^7^	Transplanted into 29 patients with chronic traumatic SCI	Perilesional intramedullary injections after thoracic and cervical SCI respectively proved safe and feasible	[151]
Human spinal cord derived-NSCs	1.2 × 10^6^	Injected into chronic spinal trauma patients	NSCs transplanted in the spinal injury site of patients can be performed safely	[153]
Human NSCs released by StemCells Inc.	1.5/3/4 × 10^7^	Transplanted into three cohorts with chronic SCI	Assessed the safety and feasibility of NSC transplants for the treated participants	[154]
Human CNS derived-NSCs	2 × 10^7^	Transplanted into phase I/IIa SCI patients	Revealed short- and long-term surgical and medical safety for NSC transplantation therapy	[155]
Human iPSC-derived NSCs/NPCs	2 × 10^6^	Transplanted into the injured spinal cord parenchyma 14–28 days post-injury of 4 patients	Assessed the safety of iPSC-derived NSC transplantation in patients and its impact on neurological function	[156]
Human CNS derived-NSCs	4 × 10^7^	Injected into 5 chronic cervical SCI patients > 4 months post-injury	Observed improvements in overall mean functional outcomes measures by 12-month clinical follow-up	[159]

## Concluding remarks and future perspectives

In summary, SCI is a complex condition that results in significant functional and anatomical changes within the spinal cord. A comprehensive understanding of the molecular, cellular and systems-level changes following SCI is essential for the development of effective therapeutic strategies to promote neural repair and functional recovery. Despite significant efforts in the field of regenerative medicine, effective treatment options for SCI remain limited. NSCs represent a promising regenerative strategy for SCI treatment due to their ability to differentiate into neurons and glial cells, of which, oligodendrocytes play a crucial role in myelin regeneration, demyelination repair and neural circuit reconstruction. In addition, the trophic factors secreted in these processes can promote tissue repair and functional recovery. Over the past decades, many preclinical studies have shown the improved motor and neural function following successful NSC transplantation in animal models of SCI. And several clinical trials tested the efficacy and safety of NSC transplantation in patients, with delightful results.

For future perspectives, although NSC transplantation holds promise for treating SCI, there are still several challenges that need to be addressed before it can be translated into clinical practice. Challenges include improving cell survival and differentiation rates, ensuring safe and effective delivery methods and uncovering the mechanisms underlying the therapeutic effects of NSC transplantation [160]. Continued studies should focus on extending cell sources, optimizing transplantation strategies and matching pre-clinical animal models with human SCI. In addition to cellular transplantation alone, combinatorial strategies based on NSCs such as co-transplantation therapies, cell delivery methods, enrichment of microenvironment, pharmacotherapeutics, biomaterial scaffolds and neurorehabilitation may have the potential to enhance injury repair following SCI [161–163]. We are confident that in the near future, NSCs have the potential to make remarkable breakthroughs in both the study of pathological mechanisms and clinical treatments for SCI.

## Data Availability

Not applicable.

## Abbreviations

SCI: Spinal cord injury
NSCs: Neural stem cells
IL-1a: Interleukin-1a
IL-1b: Interleukin-1b
IL-6: Interleukin-6
TNF: Tumor necrosis factor
CNS: Central nervous system
NPCs: Neural progenitor cells
SVZ: Subventricular zone
SGZ: Subgranular zone
OLs: Oligodendrocytes
OPCs: Oligodendrocyte precursor cells
ESCs: Embryonic stem cells
iPSCs: Induced pluripotent stem cells
FNPCs: Fetal neural progenitor cells
